# Application of analytical ultracentrifugation in gravitational sweep mode coupled with turbidity detection for analyzing polydisperse emulsions of aged biodiesel and alkanes

**DOI:** 10.1039/d5ra05601b

**Published:** 2025-12-03

**Authors:** Julian Türck, Kristian Schilling, Johannes Walter, Fabian Schmitt, Anne Lichtinger, Ralf Türck, Wolfgang Ruck, Jürgen Krahl

**Affiliations:** a Leuphana Universität Lüneburg Germany julian.tuerck@stud.leuphana.de; b Tecosol GmbH Germany; c Nanolytics GmbH Germany; d Friedrich-Alexander-Universität Erlangen-Nürnberg (FAU), Institute of Particle Technology (LFG) Germany; e Universität Hamburg, Technische und Makromolekulare Chemie Germany; f OWL University of Applied Sciences and Arts, Tecosol GmbH Germany; g Fuels Joint research Group Germany

## Abstract

Alternative fuels offer the possibility of reducing greenhouse gas (GHG) emissions with the help of the existing fleet. As a result, the introduction of new drop-in fuels leads to more complex interactions between the fuel components. Fuel aging is an important parameter in fuel design, as it describes the storage stability of the fuels. Generally, fuel aging increases polarity while simultaneously widening the polarity gap between the various fuel components. This gradient in polarity fosters the formation of emulsions, which can occur both within the fuel itself and, later, in lubricating oils (engine oils) during engine operation. The aim of this work was to find an analysis method for the investigation of emulsions of aged fuels (in this case biodiesel) and alkanes (HVO: hydrogenated vegetable oils and BO: base oil) without water or stabilizers. The difficulty here was to reproducibly measure a turbid, polydisperse and unstable system. The sedimentation velocity of HVO system was twice as high as that of the BO system. Droplet size distributions of biodiesel–HVO (BD–HVO: 500 to 5500 nm) and biodiesel–BO (BD–BO: 400 to 3750 nm) could be determined with high resolution using an analytical ultracentrifugation setup with multi-wavelength turbidity detection coupled to gravitational sweep experiments. The influence of polar molecules such as isopropylidene glycerol (solketal) and 1-octanol on the emulsions was further investigated considering the percentile size values of the respective distributions. This methodology therefore has the potential to provide a deeper understanding of the physical processes that influence the aging of engine oil.

## Abstract 2

Alternative fuels offer the possibility of reducing greenhouse gas emissions with the help of the existing fleet. As a result, the introduction of new drop-in fuels leads to more complex interactions between the fuel components.

## Graphical abstract

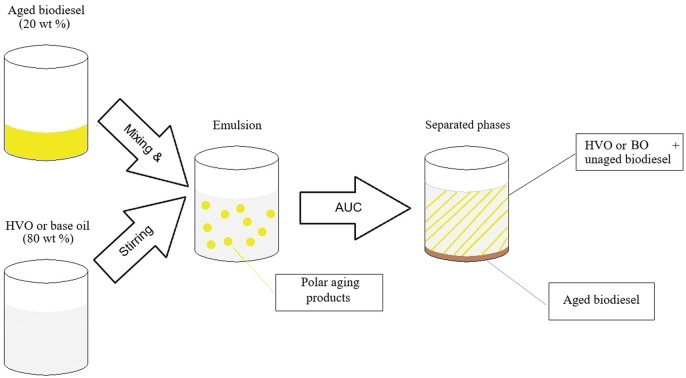

## 1 Introduction

Colloids, including emulsions, have been widely used across various industries for centuries, due to their versatile applications and unique properties. In recent years, with the help of the development of new colloidal systems and emulsions, progress has been made in the field of pharmaceuticals^1^ and in the development of new dyes and colors for high performance materials.^2,3^ Understanding the disperse properties of colloids is key for their targeted development and application. Analytical ultracentrifugation (AUC) is a powerful technique used for the precise analysis of colloids.^4^ It contributes to a better understanding of the physical and chemical properties of macromolecules and nanoparticles in solutions. AUC provides information on the size (*e.g.*, size distribution),^5^ shape (*e.g.*, conformational changes),^6^ or molecular weight of colloids,^7^ as well as particle interactions^8^ and hydrodynamic properties.^9^ Key findings included the determination of thermodynamic properties^10^ and equilibrium constants of macromolecules such as proteins and DNA^11^ as well as compositions (stochiometry) of macromolecules^12^ and approaches for multidimensional particle property characterization.^13–15^ Compared to other methods such as dynamic light scattering (DLS), laser diffraction and particle tracking analysis, AUC has far less restrictions when investigating complex colloidal systems in their natural environment. DLS^16^ and laser diffraction^17^ are limited due to polydispersity, while particle tracking analysis^18^ has lower resolution for larger and smaller particles due to small Brownian motion and low scatter signals, respectively. Due to the aging of biodiesel, polydispersity and molecular size also increase, which increasingly excludes these methods.

AUC operates on the principle of sedimentation, where particles in a sample, subjected to a centrifugal force, move towards the bottom or top of the centrifuge cell at a rate determined by their size, mass, and density. The maximum rotor speed is 60 000 rpm, which corresponds to 260 000 times the acceleration due to Earth's gravity.^19^ The resulting concentration gradients are monitored over time to obtain quantitative data from which the transport properties of the macromolecules or particles can be derived. During the process, centrifugal, frictional and buoyancy forces act on the analyte, resulting in a sedimentation velocity that can be described by sedimentation coefficients. Detecting the particles as they move in the measurement cell is crucial for assessing their sedimentation or floatation properties. Appropriate detectors are selected depending on the matrix to be analyzed. The most frequently used UV/vis detector operates according to the principle of photometry, which follows Lambert Beer's law.^20^ The measuring cell is recorded *via* consecutive scans, which allows both locally and time-resolved measurements. The extinction is measured, which is defined by the absorption and scattering of the particles in solution. However, the law has limits of validity, for example, in turbid or polydisperse inhomogeneous matrices,^16^ where the increased density/concentration of the dispersions causes the problem of multiple scattering.^21^ Depending on the samples investigated and their related optical properties, different contributions to the particle's extinction coefficient need to be considered, namely absorption, scattering or both. Another challenge that arises when analyzing large particles using AUC is that they sediment too rapidly, which can prevent accurate detection. This problem can be solved using a turbidity detector with the same technical design as a UV/vis detector, but which operates at a fixed radial position.^13,22^ In most cases, absorption can be neglected for large particles as scattering plays the predominant role.

An approach to measure broad polydisperse particles *via* turbidity detection was first described by Mäechtle,^23^ covering a particle size range from 10–3000 nm. Since the optimal rotor speed is dependent on the particle size, the gravitational sweep technique was introduced, where rotor speed is gradually increased, starting at lowest possible rates.^24^ This approach makes it feasible to determine a wide range of particle sizes in one measurement and within comparably short measurement times below 1 hour. As different particle sizes have different specific turbidities due to the size-dependent nature of scattering, this needs to be considered during data analysis. In addition, traditional turbidity detectors only allow the use of one wavelength, which limits the accessible range of concentrations to be studied and hinders identification of different materials within a single experiment.^25^ A solution for such limitations can be found in multi-wavelength UV-vis and NIR detectors, which assess particle composition- and size-dependent extinction for hundreds of different wavelengths simultaneously. Since optical densities >1 cannot be measured reliably due to non-linearity effects, the multi-wavelength option enables the measurement of turbidity at different wavelengths^26^ increasing the dynamic range of the concentrations to be studied. Size-dependent particle turbidity is calculated using Mie theory^27^ and the size distributions are then resolved using the HDR-MULTIFIT software considering all relevant wavelengths.^23,25^

By means of the turbidity detection coupled to gravitational sweep experiments, it becomes possible to measure relatively unstable emulsions *via* AUC. The field of emulsion science has gained significant relevance in the fuel sector and appears to be growing in importance. One example is emission testing of water–biodiesel emulsions, which have a high potential for emission reduction.^28^ In addition, the production of biodiesel generates glycerine, which is separated from the methyl ester by sedimentation and phase separation. In the case of more challenging feedstocks such as used cooking oil (UCO), accompanying substances can act as emulsifiers. This can lead to economically undesirable yield losses. Future biodiesel production will use increasingly waste-based feedstocks, as they are more GHG saving compared to vegetable oil based biodiesel.^29^ Emulsification and segregation tendencies also play an important role in the development of modern and complex fuels. Defossilization and renewable alkane production reduce polarity due to the absence of aromatics compared to fossil diesel fuel.^30^ In addition, polar drop-in components such as solketal are desired because they have advantages in combustion^31^ and aging behavior.^32^ Therefore, different polarity tendencies result in an increase of the polarity gap. Biodiesel plays an essential role in complex multi component fuels as it can be considered as a solubilizer for polar drop-in components due to its amphiphilic properties.^33^ However, biodiesel also has disadvantages because it contains unsaturated fatty acids that are susceptible to oxidation, known as fuel aging.^34^ The physical and chemical fuel properties change based on the incorporation of oxygen during the thermo-oxidative process.^35^

As a result, segregation and emulsion formation with regenerative alkanes occurs.^36^ This tendency is known as precipitation and shows its maximum at 20 wt% (B20) of biodiesel and is described as the B20 effect.^37^ In addition, unburned biodiesel can be described as aged and mixed with the non-polar engine oil during the engine oil dilution known for biodiesel. This question has brought the formation of emulsions with biodiesel into focus. Until then, the droplet sizes of biodiesel/water emulsions had been investigated in the literature using analytical centrifugation (LUMiSizer), microscopy and DLS.^38–40^ Due to their good atomization properties,^41^ these emulsions have a promising emission profile.^42^ However, these emulsions must be stabilized, either by surfactants or by process engineering, *e.g.*, by sonic wave treatment or homogenisators. In addition, there are studies on biodiesel emulsions in drilling fluids.^43^ This raises the question of how the physical interactions between polar aging products of biodiesel and nonpolar alkanes can be analyzed without stabilizers. In particular regarding the issue of biodiesel aging in engine oil, which leads to specific aging behavior of biodiesel due to additional interactions with additives (*e.g.* with Fe(iii) or zinc dialkyldithiophosphates).^44–47^ In this paper, a methodology was developed based on AUC with multi-wavelength turbidity detection coupled to gravitational sweep experiments to study the interaction between polar aging products and alkanes by means of the size distribution of the formed emulsion droplets. Compared to other emulsion studies, this work represents a step towards investigating the interaction of fuel emulsions without water and stabilizers. The special configuration was chosen because the relative instability of the emulsion requires the gravitational sweep mode and the limitation of detection necessitated the multi-wavelength absorption (MWA) detector. This represents a novelty, as no steps have yet been taken to quantify the interactions of fuel/fuel emulsions using AUC. We show the reproducible measurement of droplet sizes of emulsions of aged biodiesel with both hydrogenated vegetable oils (HVO) and base oil (BO; non-additive engine oil). Furthermore, 1-octanol and solketal were added to the investigated emulsions to gain a deeper insight into the emulsification process when adding polar molecules. The investigations indicate that fuel/fuel emulsions contribute significantly to fuel aging, which means that this problem has to be addressed more intensively.

## 2 Experimental section

### 2.1 Chemicals

The fuels used, biodiesel and HVO, were purchased from Analytik Service Gesellschaft AG, Germany. The biodiesel used is based on rapeseed oil and therefore has a typical fatty acid composition of this vegetable source. The fuel analysis of the two components is listed in the SI and was determined by Analytik Service Gesellschaft AG, Germany. The BO is a category 3 oil designated Yubase 4 (CAS: 64742-54-7) supplied by TotalEnergies Societas Europaea, France. The polar additives were 1-octanol and solketal. Both chemicals were purchased from Merck KGaA, Germany. Solketal had a purity of 98% and 1-octanol of >99%.

### 2.2 Fuel aging

Fuel aging was performed according to EN 14112 by using a rancimate. A detailed experimental setup of the rancimate was described by Türck *et al.*^48^ The fuel that was subjected to aging was biodiesel. For this purpose, 20 g of biodiesel were subjected to the rancimate at a temperature of 110 °C. The applied air flow was 166.7 mL min^−1^. The electrical conductivity of the decomposition products was measured in the eluate (50 mL distilled water). The aging time of the biodiesel was 40 hours.

### 2.3 Preparation of the emulsions

**Fig. 1:**
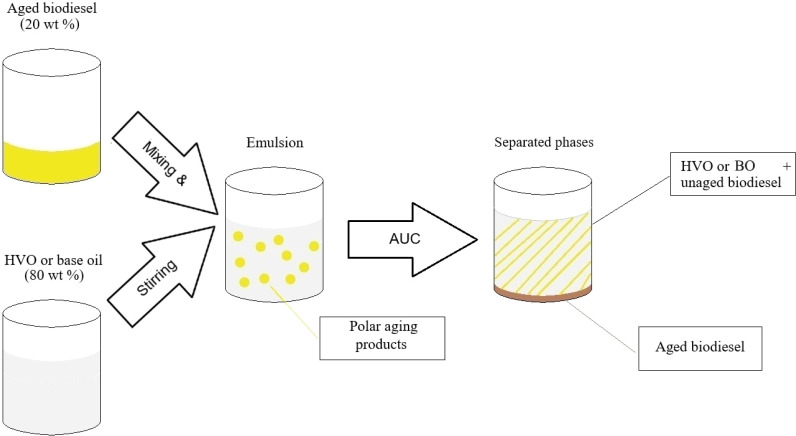
Formation of the emulsion by adding 20 wt% biodiesel and 80 wt% hydrocarbons (HVO or BO). Polar aging products are involved in the formation of the emulsions together with the hydrocarbons. Phase separation is accelerated by AUC, resulting in two phases: the hydrocarbons + unaged biodiesel in the upper phase and the aged biodiesel in the lower phase.

The emulsions were freshly prepared prior to each measurement. The formation of the emulsion is highlighted in Fig. 1. For this purpose, 20 wt% of the fuel components (aged biodiesel) were stirred with 80 wt% of the alkane fractions (HVO and BO) in a beaker for 5 minutes. This proportion was determined based on the B20 effect. After preparation and stirring, the emulsion was measured immediately. All emulsions were stirred under constant conditions to eliminate mixing as an additional parameter for the emulsification process. Polar additives were introduced into the two analyzed emulsion systems to evaluate their effects on the emulsion. For the BD–HVO system, 2–8 wt% solketal (based on the total quantity) was provided in 2 wt% increments. For the BD–BO system, 5–20 wt% 1-octanol was added in 5 wt% increments. After brief stirring and vortexing for 5 minutes, the emulsions were transferred to measuring cells equipped with titanium centerpieces (12 mm). The volume was kept constant.

### 2.4 AUC experiments and data analysis

AUC measurements were carried out at Nanolytics GmbH, Germany. The sedimentation experiments were performed with two LE-80k ultracentrifuges from Beckman Coulter equipped with MWA detectors from Nanolytics Instruments.^49^ This detector can be used for localized turbidity observation. The experiments were carried out in gravitational sweep mode, where the speed was continuously increased. Detection took place from a rotor speed of approx. 1000 rpm, whereby complete sedimentation was observed at approx. 4000 rpm. In addition, the detector can record radial extinction profiles at a fixed rotor speed to determine the meniscus position and phase separation of the emulsion after the gravitational sweep experiment. The gravitational sweep measurements were taken at radius of 6.5 cm. Only the solketal measurements were taken at a position of 6.6 cm to allow the emulsion droplets more time to pass the measuring point. The meniscus position and phase boundary after segregation were determined using a radial scan after completing the measurement. The temperature was 20 °C for all measurements. From the available data of the UV-vis spectrum, the wavelength of 400 nm was selected for data analysis. Turbidity data were analyzed using HDR-MULTIFIT^25^ and converted into mass-weighted particle size distributions. The software additionally calculated the percentile size values of the distributions. Table 1 describes the parameters applied for the evaluation of the emulsions. The density of dispersant and the refractive index of the dispersion medium was determined after separation of the emulsion.

**Table 1:** Physical parameters for both emulsions to assess the droplet size ranges

Emulsion	BD–HVO	BD–BO
Density of the dispersion medium [g mL^−1^]	0.76	0.83
Density of the dispersant [g mL^−1^]	0.96	0.96
Refractive index of the dispersion medium	1.45	1.45
Refractive index increment of the dispersant	0.03	0.04

**Fig. 2:**
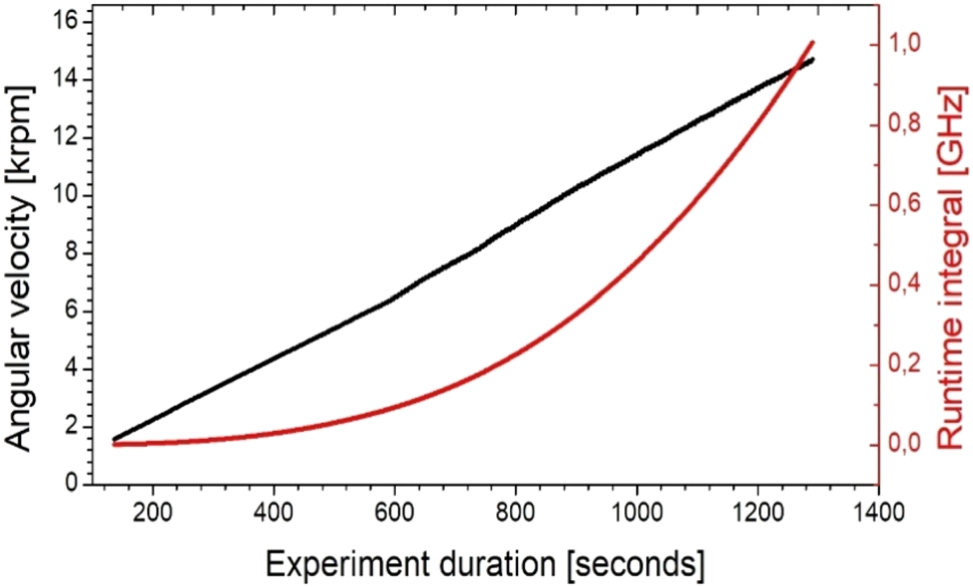
The change in the angular velocity of the rotor during the duration of the experiment using the gravitational sweep method. The runtime integral (a measure for the total centrifugal force the particles have been exposed to until the given time) is displayed in red.

The contribution of solketal to the overall density was not taken into account for the calculation of the droplet sizes. The larger density of solketal (1.063 g mL^−1^) is mitigated in its effect on the determined emulsion droplet diameters from the derived sedimentation coefficients, since firstly only a maximum of 8 wt% is present and secondly the density differences are only included in the conversion as square roots. In general, changing the concentration of the emulsion components can be a limiting factor, as this can lead to excessive differences in density. This can result in a lack of comparability. The evolution of angular velocities during the gravitational sweep experiments is shown in Fig. 2. The experiments with 1-octanol started at a rotor speed of 1000 rpm.

## 3 Results and discussion

### 3.1 General procedure and observation

**Fig. 3:**
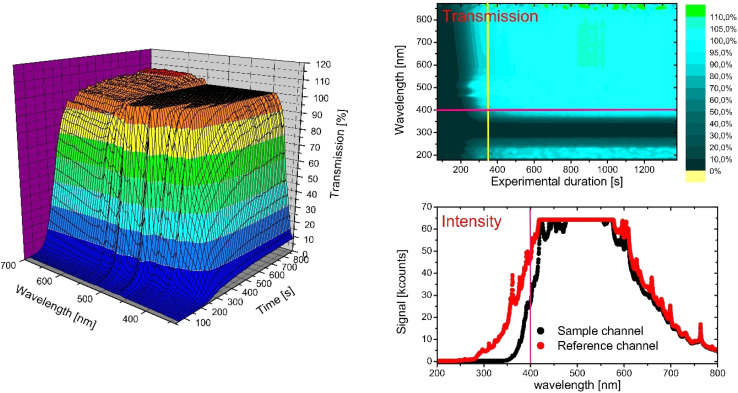
Left: 3D diagram to illustrate a topographical representation of the relationships between transmission, wavelength and experimental duration. Top right: 2D heat map of transmission to demonstrate the selection of wavelength (pink bar, 400 nm) for a specific experimental duration (yellow bar, 350 seconds). Bottom right: Typical lamp spectrum at 350 seconds with a red bar at 400 nm. Red indicates the reference channel, while black indicates the sample channel.

Initially, the transmission spectra of both emulsions were recorded over the entire measurement duration using an MWA detector (Fig. 3). In the following section, insights into the data processing strategy will be provided. The 3D plot in Fig. 3 demonstrates the process of selecting the most useful wavelength segment from the data provided by the multi-wavelength detector. The topographic representation on the left shows sample transmission (sample intensity divided by reference intensity) as a function of time and wavelength. The flat upper plateau indicates a cutoff due to detector saturation, unlike the region towards longer wavelengths, which exhibits some noise. The most useful data, however, is found at the front right slope indicating lower wavelengths, where transmission rises closely to 100% but over a longer stretch of time – representing the entire sedimentation process. A more abstract data representation is given as a 2D plot in the top right panel of Fig. 3, yielding more detailed insights. Basically, a top view of the left panel, where the third dimension is represented by colors, visualizes increasing transmission by blue shades progressing from dark to light. Erroneous values outside the range between 0 and 100% (due to calculation artifacts for extremely high and low intensity readings) are obvious as green and yellow pixels. At a first view, it is apparent that there was no transmission in a range between 250 and 350 nm, rendering this data range unfit for evaluation. A closer inspection of a typical intensity spectrum, taken from the upper plot at an experimental duration of 350 seconds (as indicated by a vertical yellow line), shows that this is due to zero transmission in the sample below 350 nm, though the reference allowed for some transmission. However, below 280 nm, the reference transmission dropped to zero as well. Whilst the 2D plot may suggest some useful transmission in this range, the bottom plot clearly shows that these values originate from division of values close to zero intensity. In this manner, a combination of different data representations allows for discrimination of useful and non-useful data. Further conclusions are that between 480 and 575 nm, the reference channel generated such high intensities that the detector was saturated, as seen before in the 3D plot. Data above 600 nm were not useful because there was only a small difference between sample and reference signal due to low extinction values, providing transmission close to 100% regardless of sample concentration. Data between 350 and 380 nm provided a large difference between sample and reference signal, but the lamp signal was too low for a reliable analysis. The optimal range is slightly above this range, and a wavelength of 400 nm was chosen (marked as pink horizontal line in the 2D plot) as most appropriate as it provided high lamp signals, strong particle extinction, and a good exploitation of the transmission range between 0 and 100%.

### 3.2 Biodiesel–HVO/BO emulsions

**Fig. 4:**
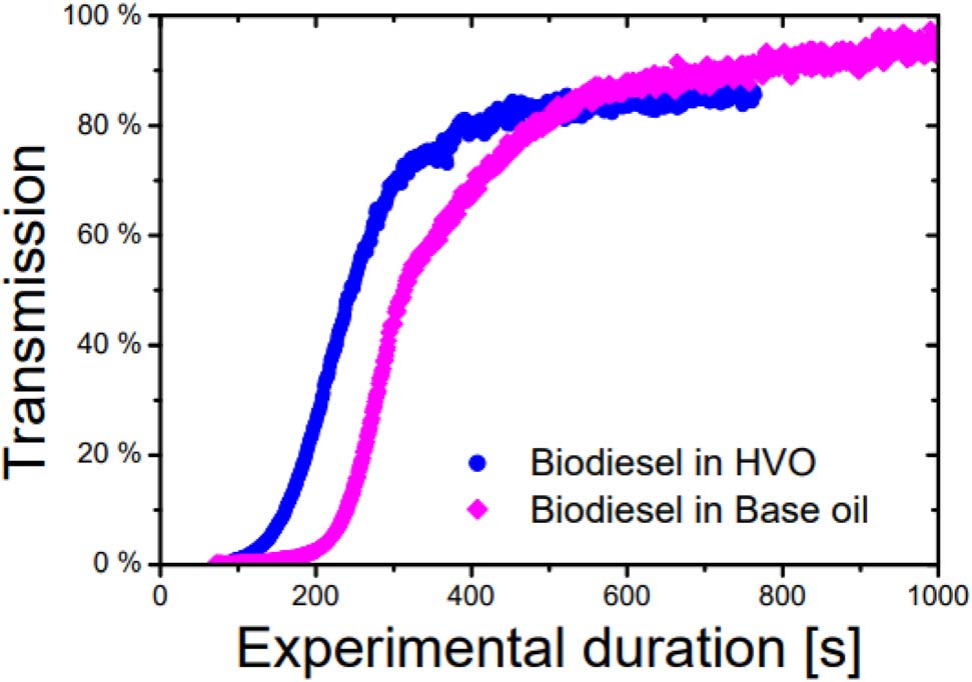
Time progression of transmission raw data for the BD–HVO emulsion system (blue) and the BD–BO system (pink) during AUC measurement.

**Fig. 5:**
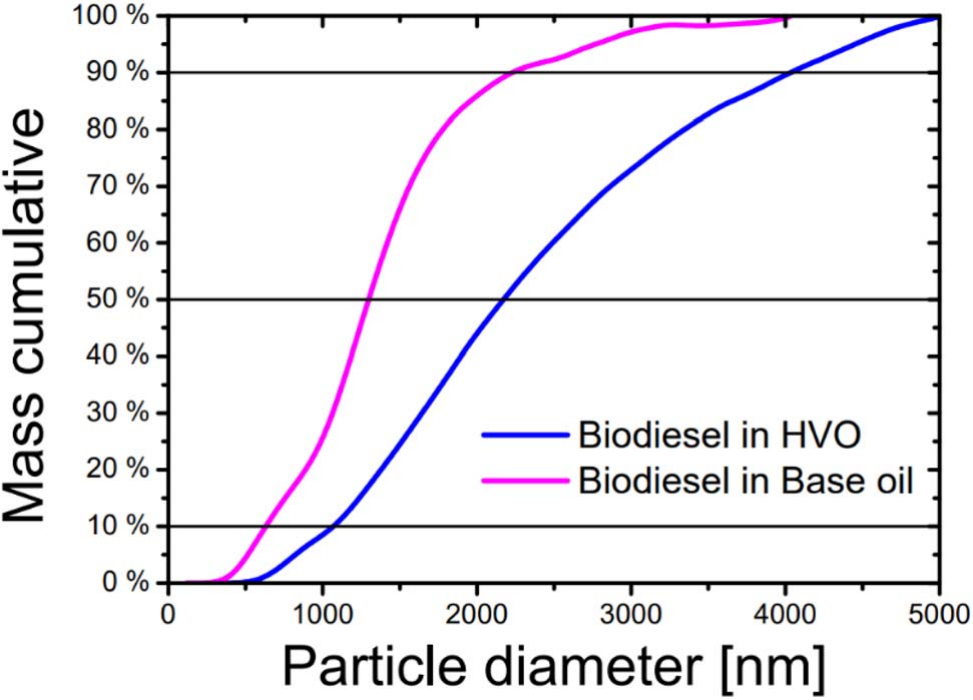
Mass-weighted cumulative droplet size distribution of the emulsion systems with the aged biodiesel and HVO (blue) and BO (pink), measured in gravitational sweep mode with a turbidity detector. The black line shows the determination of the percentiles size values based on particle mass (10% *d*_10_, 50% *d*_50_ and 90% *d*_90_).

In principle, the challenge was to establish a reproducible method for both systems despite their polydispersity and rapid sedimentation. One of the reasons for this is due to the difference in density between aged biodiesel and the alkane fraction. This was corroborated by the fact that the sedimentation velocity in HVO was twice as high as in BO system (Fig. 4). This is, on the one hand, due to the lower density difference between aged biodiesel and BO (0.13 compared to 0.2 g mL^−1^) and, on the other hand, due to the different particle sizes. Furthermore, the polydispersity of each of the two phases leads to a statistically broad distribution of possible interactions, causing different droplet sizes. In addition, biodiesel forms molecules of different sizes during fuel aging, which further increases the possibility of different emulsion droplets forming. This was evident in the repeat measurements, in which the shape of the distributions varied for the two systems. However, it was possible to reproduce the range of droplet sizes. Fig. 5 depicts the mass cumulative droplet size distributions for both the HVO and the BO system.

**Fig. 6:**
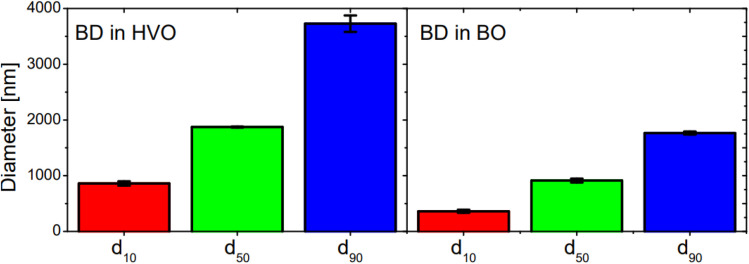
Bar chart of the percentile values *d*_10_ (red), *d*_50_ (green) and *d*_90_ (blue) of the BD–HVO and BD–BO system with illustration of the reproducibility. The reproducibility is determined by preparing and measuring different emulsions (*n* = 3).

In the HVO system, the droplets were found in a range between 500 and 5500 nm. In the BO system, the range was also reproducible and varied between 400 and 3750 nm. However, a sharper distribution could be observed here, since the main particle sizes were between 900 to 2000 nm. This was also evident from the steeper cumulative distribution for BO compared to HVO. This could indicate a greater orientation of the aging products in the BO matrix. A possibility could relate to the different chemistry of the alkane fractions. In terms of the various boiling ranges of mineral oil, HVO is orientated towards the diesel boiling range and contains hydrocarbons with a chain of C from 16 to 18.^50^ In principle, BOs are in the lubricating oil fraction of crude oil and have a carbon chain from C_18_ to C_34_.^51^ This steric difference of the alkanes could result in a stronger orientation of the polar aging product of the biodiesel in the non-polar matrix. Comparable observations of the steric influence of emulsion formation have been addressed in several works.^52,53^ Another way to validate the reproducibility of the method and make different measurements comparable was to examine the percentile droplet size values *d*_10_, *d*_50_ and *d*_90_. They describe the mass percentages of droplets that are smaller than the specified percentile value. This means that at *d*_10_ 10%, at *d*_50_ 50% and at *d*_90_ 90% of the particles in terms of mass are smaller than the given size. Fig. 6 displays the percentile values of the two systems.

The error bars of both systems confirm reproducibility of the measurements. The fluctuations in the errors are smaller in the BO system than in the HVO system which is particularly evident for the *d*_90_ value. This might also indicate that larger droplets in the HVO system tended to be less stable. Furthermore, a statistical trend can be deduced from the droplet sizes of the two emulsions. The HVO system produced larger droplets compared to the BO system (40–50% for the three percentile values). Another important parameter for characterizing emulsions is their polydispersity in size. The polydispersity index (PDI) can be determined using the equation (*d*_90_ − *d*_10_)/*d*_50_ and remained approximately the same for both systems (BD–HVO: 1.53 ± 0.07, BD–BO: 1.54 ± 0.1).

### 3.3 Influence of polar molecules

**Fig. 7:**
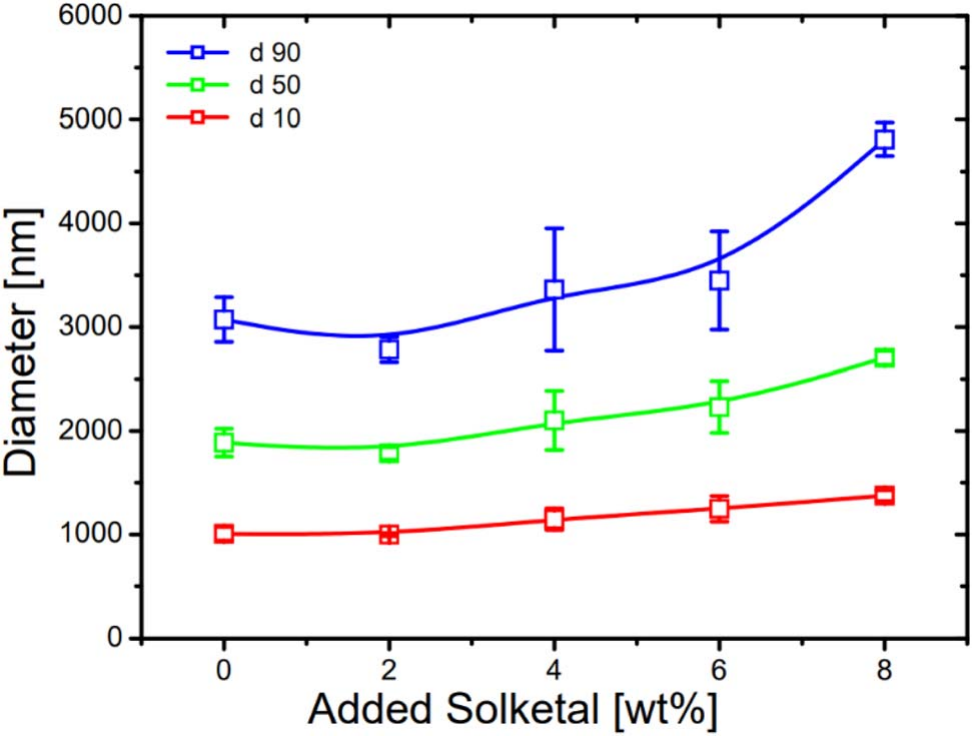
Progression of the percentile mass-weighted particle sizes *d*_10_ (red), *d*_50_ (green) and *d*_90_ (blue) with increasing solketal content (wt%) (*n* = 3).

After it was demonstrated that AUC operated in gravitational sweep mode and combined with turbidity detection can be used for a reproducible investigation of the emulsion droplet size distribution of aged biodiesel–alkane emulsions, the influence of polar molecules on the system was investigated in the subsequent section. On the one hand, the change in polarity can lead to greater phase separation or larger/smaller formation of emulsions, as some of the polar aging products tend to be soluble in the polar phase. On the other hand, the polar molecules can act as emulsion breakers and thus form fewer or no more droplets. For this purpose, solketal was added to the HVO and 1-octanol to the BO system, as described in the Experimental section. Due to the higher molecular oxygen density, solketal is more polar compared to 1-octanol. This increases the potential for increasing the volume of the polar phase, including solketal. 1-Octanol, however, is characterized by its amphiphilic properties.^54^ The course of the percentile mass-weighted particle sizes with increasing solketal content is shown in Fig. 7.

The addition of solketal caused the size distribution to shift towards larger diameters, which was reflected in larger values for *d*_10_, *d*_50_ and *d*_90_ (see Fig. 7 and Table 2). Furthermore, the error bars indicate that the addition of solketal increased the instability of the emulsions as the reproducibility decreases. The polydispersity of the samples was approximately the same (0.99 to 1.09) when up to 6 wt% solketal was added. In addition, it appears that solketal addition tends to form larger droplets, because the increase was greater at *d*_90_ compared to *d*_50_ (36% *vs.* 30%) and *d*_10_ (36% *vs.* 27%). For a more accurate absolute determination of droplet sizes in the presence of solketal, the density of the dispersed phase must always be determined.

**Table 2:** List of percentile mass-weighted particle sizes for different solketal contents in the BD–HVO system

Solketal content [wt%]	*d*_10_ [nm]	*d*_50_ [nm]	*d*_90_ [nm]
0	1006	1886	3071
2	998	1783	2782
4	1147	2101	3361
6	1248	2229	3447
8	1375	2702	4809

**Fig. 8:**
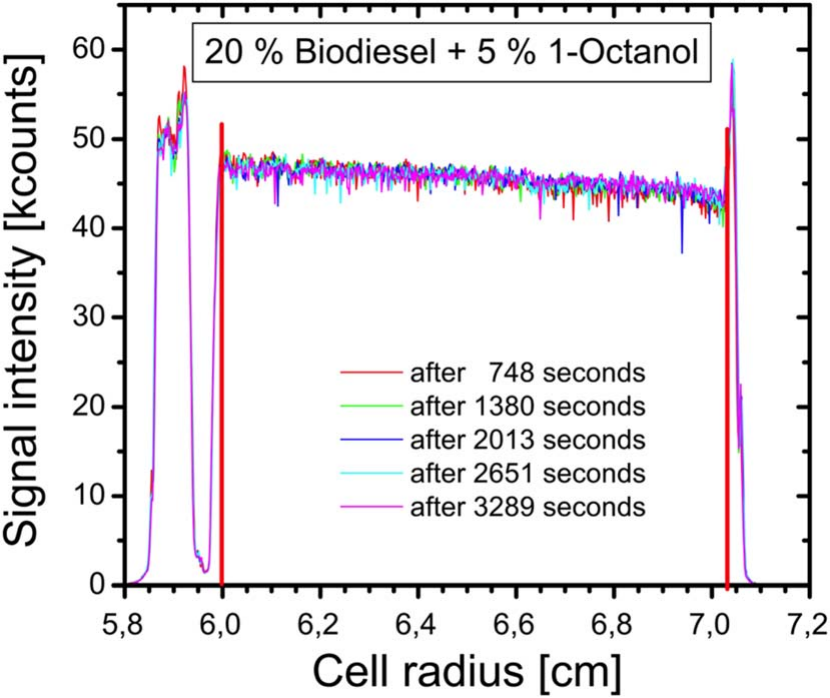
Exemplary plot for a locally and time resolved SV-AUC measurement of the BD–BO system with 5 wt% 1-octanol using a constant rotor speed of 1000 rpm. It produces concentration profiles along the radial coordinate (distance from the axis of rotation). The different colors represent the various measurement times. The red lines show the meniscus (left) and the cell floor (right).

The measuring cells were submitted to a radial scan after completion of the measurement to confirm that segregation had occurred. The radial scans are displayed in the SI. It is evident that there was no significant phase boundary shift with increased solketal addition (see Table 3). This suggests that the increased droplet sizes were not due to a larger polar phase. However, the increasing droplet sizes indicate that solketal plays a role in the emulsion formation process. Solketal itself is a surface-active component, which exhibit a higher surface tension compared to other fuels.^55^ It is conceivable that this surface activity promotes intermolecular interaction and can lead to an emulsion formation. In contrast, measurements with 1-octanol in the biodiesel–BO matrix revealed at different behavior as no sedimentation was observed during the gravitational sweep experiments. Radial measurements were performed for verification purposes. Fig. 8 shows as an example the emulsion with 5 wt% 1-octanol. All other measurements can be seen in the SI. It can be observed that measured signal was constant between the meniscus (left red line in Fig. 8) and the cell floor (right red line in Fig. 8) and that this did not change over time. Furthermore, the radial scans showed that a homogeneous phase was formed and that there was no phase boundary observable. Hence, it can be concluded that no emulsion particles were formed between 5 and 20% 1-octanol, which confirms that 1-octanol serves as an emulsion breaker. It also shows that the addition of a polar molecule can either break, stabilize or promote the formation of an emulsion depending on its composition and concentration.

**Table 3:** Average radial position (with error) of the phase boundaries inside the measuring cell for different solketal contents (*n* = 3)

Solketal content [wt%]	Average phase boundary position [cm]	Error [cm]
0	7.05	0.01
2	7.04	0.01
4	7.00	0.07
6	6.94	0.08
8	7.00	0.05

The enlargement of the droplets by solketal should occur due to a higher viscosity of the dispersed phase^55^ and dilution of the surfactant at constant surfactant to oil ratio (SOR). In combination, they suppress interfacial area formation during high-energy emulsification, in line with findings that greater solketal fractions produce larger droplets and higher emulsion viscosity,^56,57^ as well as with general emulsification theory.^52^ In contrast, traces of 1-octanol localize at the interface and enhance interfacial mobility. The resulting drop in interfacial elasticity and surface viscosity weakens Gibbs–Marangoni stabilization and accelerates coalescence. 1-Octanol's demulsifying behavior in oil–water systems are well documented^58^ and is mechanistically consistent with the micro segregated (reverse-micelle-like) organization of wet octanol,^59^ which rapidly rearranges under shear and interfacial geometry.^54^

In terms of practical application, this work shows that biodiesel formulations should aim for a balance between viscosity and density of the phases to enable separation without promoting stable emulsions. Consequently, impurities, water, and other polar species should be minimized, and co-solvents should be added only at levels that do not raise emulsion risk. Due to the relative instability of these emulsions, these influencing factors are crucial in terms of fuel or engine oil aging. Nevertheless, emulsions can be formed between alkanes and polar aging products of biodiesel, which makes additional breakers such as 1-octanol worth considering.

## 4 Conclusion and outlook

In this study, it was demonstrated that it is possible to utilize an AUC with a turbidity detector in gravitational sweep mode for size characterization of biodiesel/alkane emulsions. Despite the rapid sedimentation and high polydispersity of the systems of aged biodiesel with alkanes, they could be analyzed in terms of their droplet size distributions. This work therefore represents a further step toward a better understanding of the physical interactions between the polar aging products of biodiesel and the alkane phases without stabilizers. It holds the promise of being another step toward a better understanding of the complex aging of biodiesel.

The density and the molecular sizes of the alkanes defined how quickly the segregation took place. The chemical composition of the alkanes then determines the droplet size range (BD–HVO: 500–5500 nm, BD–BO: 400–3750 nm). Furthermore, the alkanes appeared to influence the orientation and steric properties of the droplets, which was evident in the comparatively sharper distribution of the BO. The addition of polar molecules demonstrated the occurrence of two phenomena. On the one hand, the increasing addition of solketal to the BD–HVO system indicated an increase for larger particles. On the other hand, polar molecules could also help to break the emulsions, as can be seen in the example of 1-octanol in the BD–BO system.

Now that a method for investigating these emulsion systems had been established, complementary methods should be found to verify the identified droplet size ranges. Moreover, further physical parameters need to be analyzed in the next steps. The focus should be on parameters that influence agglomeration and phase separation, such as temperature. In addition, mechanical influences on the dispersion process that affect the specific energy input for the emulsion droplet formation must be investigated. This allows an evaluation of whether the droplets enter into new interactions under certain mechanical, physical or chemical conditions. Furthermore, this could lead to a deeper understanding of the stability criteria and the phase separation kinetics. In general, further steps must be taken into account for additional restrictions, as the methodology is limited in terms of droplet size. This means that larger aggregates that may form cannot be examined. However, investigating the interaction of polar aging products in emulsions offers the opportunity to gain a more detailed knowledge of the aging mechanisms in engine oil and fuel and can therefore address the topic of engine oil dilution. It has the potential to contribute to understanding the exact aging process of engine oil. This could increase the efficiency of lubricating oils and at the same time enable a higher biodiesel content, which is currently limited due to the dilution of the engine oil.

## Conflicts of interest

There are no conflicts to declare.

## Supplementary Material

RA-015-D5RA05601B-s001

## Data Availability

The data supporting this article have been included as part of the supplementary information (SI). Supplementary information is available. See DOI: https://doi.org/10.1039/d5ra05601b.
