# Correction: Application of analytical ultracentrifugation in gravitational sweep mode coupled with turbidity detection for analyzing polydisperse emulsions of aged biodiesel and alkanes

**DOI:** 10.1039/d5ra90143j

**Published:** 2025-12-17

**Authors:** Julian Türck, Kristian Schilling, Johannes Walter, Fabian Schmitt, Anne Lichtinger, Ralf Türck, Wolfgang Ruck, Jürgen Krahl

**Affiliations:** a Leuphana Universität Lüneburg Germany julian.tuerck@stud.leuphana.de; b Tecosol GmbH Germany; c Nanolytics GmbH Germany; d Friedrich-Alexander-Universität Erlangen-Nürnberg (FAU), Institute of Particle Technology (LFG) Germany; e Universität Hamburg, Technische und Makromolekulare Chemie Germany; f OWL University of Applied Sciences and Arts Germany; g Fuels Joint Research Group Germany

## Abstract

Correction for “Application of analytical ultracentrifugation in gravitational sweep mode coupled with turbidity detection for analyzing polydisperse emulsions of aged biodiesel and alkanes” by Julian Türck *et al.*, *RSC Adv.*, 2025, **15**, 47840–47849, https://doi.org/10.1039/D5RA05601B.

The authors regret that affiliation *f* was incorrect in the original manuscript. The correct affiliation is as shown herein.

The Royal Society of Chemistry apologises for these errors and any consequent inconvenience to authors and readers.

