# Supplementary material for: Application of analytical ultracentrifugation in gravitational sweep mode coupled with turbidity detection for analyzing polydisperse emulsions of aged biodiesel and alkanes
Source: RSC Adv. 2025 Dec 3;15(56):47840–9. doi: 10.1039/d5ra05601b (PMC12679377; doi:10.1039/d5ra05601b)
Supplement: RA-015-D5RA05601B-s001 [file RA-015-D5RA05601B-s001.pdf]

Supplementary Information (SI) for RSC Advances.
This journal is © The Royal Society of Chemistry 2025

Supporting Information

For

Application of analytical ultracentrifugation in gravitational sweep mode coupled with turbidity detection for analyzing polydisperse emulsions of aged biodiesel and alkanes

Julian Türck,*[a,d] Kristian Schilling,[b] Johannes Walter,[c] Fabian Schmitt,[d] Anne Lichtinger,[e] Ralf Türck,[d,f] Wolfgang Ruck[a] and Jürgen Krahl[e,f]

[a] Leuphana Universität Lüneburg, Universitätsallee 1, 21335 Lüneburg, Germany

[b] Nanolytics GmbH, Am Mühlenberg 11, 14476 Potsdam, Germany

[c] Friedrich-Alexander-Uiniversität Erlangen-Nürnberg, Cauerstraße 4, 91058 Erlangen, Germany

[d] Tecosol GmbH, Jahnstraße 2, 97199 Ochsenfurt, Germany

[e] OWL University of Applied Sciences and Arts, Campusallee 12, 32657 Lemgo, Germany

[f] Fuels Joint Research Group, www.fuels-jrg.de, Germany

Fuel analysis

HVO

**Test parameter**	**Test method**	**Test result**	**Limit value DIN EN 15940 :2019**		**Unit**
			**min.**	**max.**	
Cetane number (ICZ)	DIN EN 17155 :2018	74.8	-	-	-
Density (15°C)	DIN EN ISO 12185 :1997	780.5	765	800	mg/m³
Flash point	DIN EN ISO 2719 : 2021	74.5	>55	-	°C
Kin. Viscosity (40°C)	DIN EN ISO 3104 : 2021	2.973	2.00	4.50	mm²/s
Volume at 250°C	DIN EN ISO 3924 : 2019	4.5	-	<65	% (V/V)
Volume at 350°C		-	85	-	% (V/V)
95 % point		296.0	-	360	°C
HFRR (lubricity at 60 °C)	DIN EN ISO 12156-1 :2019	432	-	460	µm
Fatty acid methyl ester content	DIN EN 14078 :2014	<0,01	-	7.0	% (V/V)
Manganese (Mn)	DIN EN 16576: 2015	<0,50	-	2.0	mg/l

Total aromatics	DIN EN 12916 :2022 Verf. B	<0,1	-	1.1	% (m/m)
Sulphur content	DIN EN ISO 20884: 2022	<5(<1)	-	5.0	mg/kg
Coke residue (10%D.)	DIN EN ISO 10370 :2015	<0,1	-	0.30	% (m/m)
Ash content (775°C)	DIN EN ISO 6245 :2003	<0,001	-	0.01	% (m/m)
Water content	DIN EN ISO 12937: 2002	<30	-	200	mg/kg
Total pollution	DIN EN 12662 :2014	<12	-	24	mg/kg
Corrosion effect on copper	DIN EN ISO 2160 :1999	1	-	Class 1	Corr. Degree
Filterable Ageing residue	DIN EN ISO 12205 :1996	<1	-	-	g/m³
Unfilterable Ageing residue		<1	-	-	g/m³
Oxidation stability		<1	-	25	g/m³
Oxidation stability	DIN EN 15751 :2014	-	20	-	h
CFPP	DIN EN 116 :2018	-24	-	*	°C
Calorific value (Hu,p)	DIN 51900-2 :2003 mod.	43.6	-	-	MJ/kg
Carbon content	DIN 51732 : 2014	84.84	-	-	% (m/m)
Hydrogen content		15.41	-	-	% (m/m)
Nitrogen content		<0,5	-	-	% (m/m)

Biodiesel

**Test parameter**	**Test method**	**Test result**	**Limit value DIN EN 14214 _ 2014** min.	max.	**Unit**
BHT	ASG 1803-GC-FID	<50	-	-	mg/kg
C6:0 / capyric acid	DIN EN 14103 :2015	<0,1	-	-	% (m/m)
C8:0 / caprylic acid		<0,1	-	-	% (m/m)
C10:0/ Capric acid		<0,1	-	-	% (m/m)
C12:0/ lauric acid		<0,1	-	-	% (m/m)
C14:0 / myristic acid		<0,1	-	-	% (m/m)
C16:0/ Palmitic acid		4.7	-	-	% (m/m)

C16:1 / palmitoleic acid		0.3	-	-	% (m/m)
C17:0/ Heptadecanoic acid		<0,1	-	-	% (m/m)
C18:0 / stearic acid		1.7	-	-	% (m/m)
C18:1 / oleic acid		63.2	-	-	% (m/m)
C18:2 / linoleic acid		19.1	-	-	% (m/m)
C18:3 / linolenic acid		8.0	-	-	% (m/m)
C20:0 / arachidic acid		0.6	-	-	% (m/m)
C20:1 / gadoleic acid		1.1	-	-	% (m/m)
C22:0 / Behenic acid		0.3	-	-	% (m/m)
C22:1 / erucic acid		0.1	-	-	% (m/m)
C24:0 / Lignoceric acid		<0,1	-	-	% (m/m)
C24:1 / Nervonic acid		<0,1	-	-	% (m/m)
Unknown fatty acids		0.6	-	-	% (m/m)
Ester content		>99	96.5	-	% (m/m)
Linolenic acid ME content		8.1	-	12.0	% (m/m)
Density (15°C)	DIN EN ISO 12185 :1997	881.3	860	900	kg/m3
Kin. Viscosity (40°C)	DIN EN ISO 3104 :2021	4.256	3.50	5.00	mm2/s
Flash point	DIN EN ISO 2719 :2021	186.5	101	-	°C
CFPP	DIN EN 116 :2018	-9	-	*	°C
Sulphur content	DIN EN ISO 20884: 2022	<5(1,3)	-	10	mg/kg
Cetane number (ICZ)	DIN EN 17155 :2018	51.7	51.0	-	-
Sulphated ash (775°C)	ISO 3987 :2010	<0,01	-	0.02	% (m/m)
Water content	DIN EN ISO 12937 :2002	40	-	500	mg/kg
Total pollution	DIN EN 12662 :1998	<1	-	24	mg/kg
Corrosion effect on copper	DIN EN ISO 2160 :1999	1	-	1	Corr. degree
Oxidation stability	DIN EN 14112 :2021	1.8	8.0	-	h
Acid value	DIN EN 14104 :2003	0.24	-	0.50	mg KOH/g
Iodine number	DIN EN 16300 :2012	110.2	-	120	g Iod/100 g
PUFA	DIN EN 15779 :2013	<0,60	-	1.00	% (m/m)
Methanol content	DIN EN 14110	<0,01	-	0.20	%

	:2019				(m/m)
Free glycerol content	DIN EN 14105 :2011	0.008	-	0.02	% (m/m)
Monoglyceride content		0.11	-	0.70	% (m/m)
Diglyceride content		<0,01	-	0.20	% (m/m)
Triglyceride content		<0,01	-	0.20	% (m/m)
Total glycerol content		0.037	-	0.25	% (m/m)
Alkali content (Na+K)	DIN EN 14538 :2006	<1	-	5.00	mg/kg
Alkaline earth content (Ca+Mg)		<1	-	5.00	mg/kg
Phosphorus content	DIN EN 14538 :2003	<4 (<0,5)	-	4.00	mg/kg
Cloudpoint	DIN EN 23015 :1994	-8	-	*	°C

Radial Scans

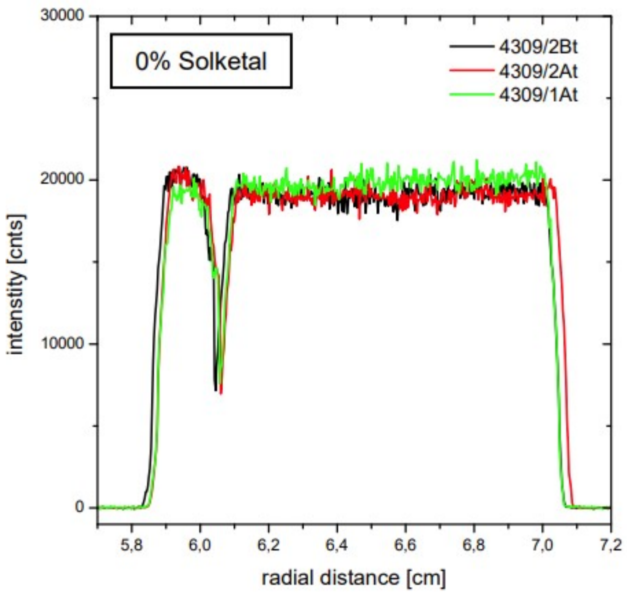

Figure 1*: Radial scan of the measuring cell to determine the phase boundary position (0 wt% solketal). The different colors describe different experiments (n=3).*

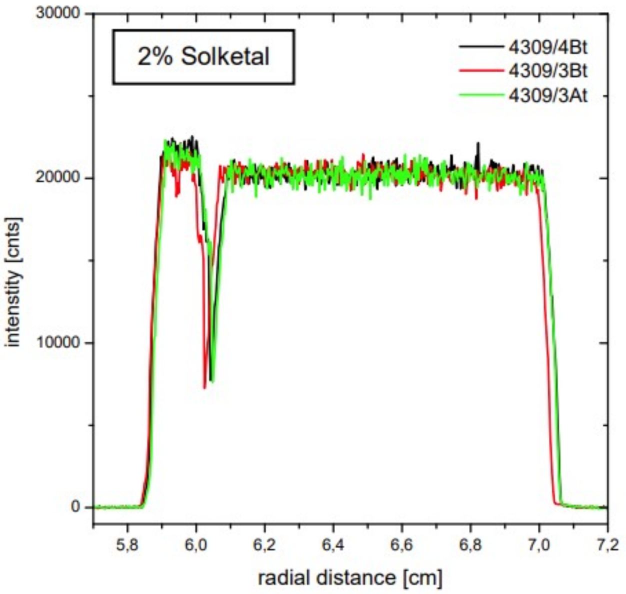

Figure 2: *Radial scan of the measuring cell to determine the phase boundary position (2 wt% solketal). The different colors describe different experiments (n=3).*

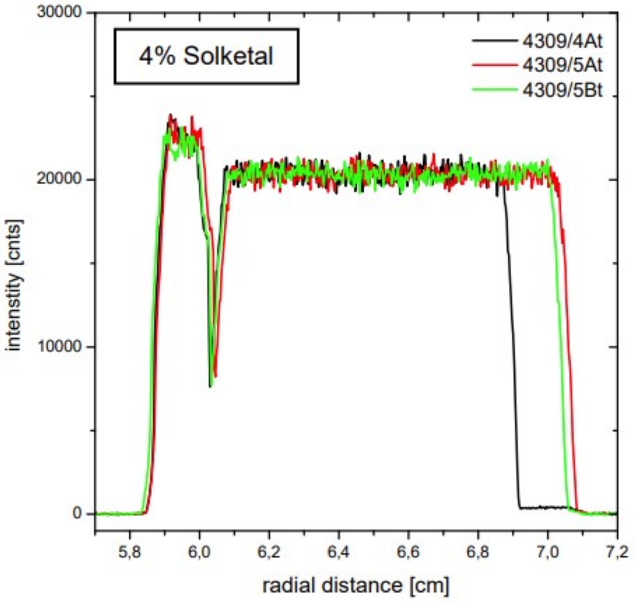

Figure 3: *Radial scan of the measuring cell to determine the phase boundary position (4 wt% solketal). The different colors describe different experiments (n=3).*

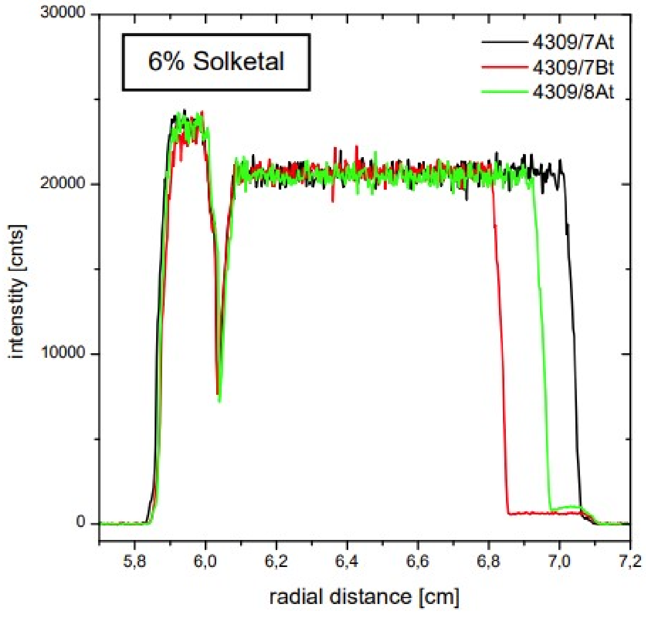

Figure 4: *Radial scan of the measuring cell to determine the phase boundary position (6 wt% solketal). The different colors describe different experiments (n=3).*

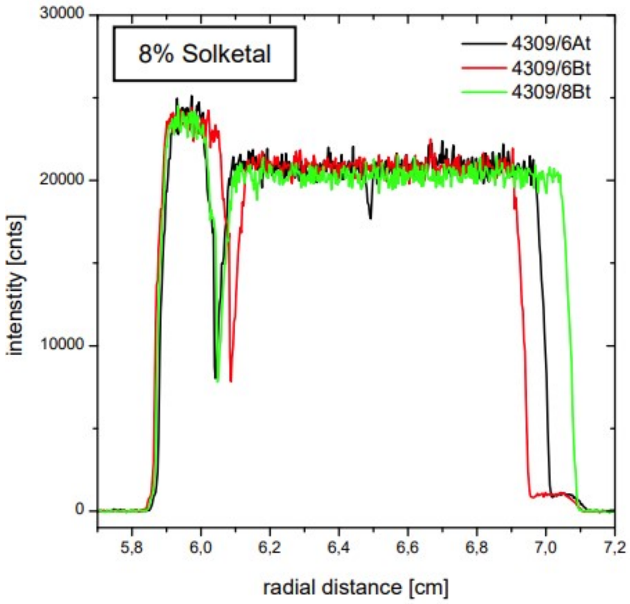

Figure 5: *Radial scan of the measuring cell to determine the phase boundary position (8 wt% solketal). The different colors describe different experiments (n=3).*

SV-AUC BD-BO with 1-octanol

BO + 10 % 1-octanol

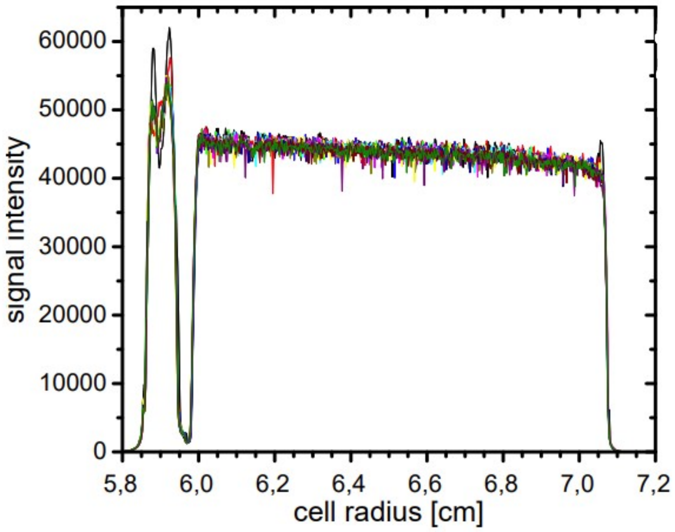

Figure 6: *SV-AUC measurement of the BD-BO system with 10 wt% 1-octanol using a constant rotor speed of 1000 rpm. It produces concentration profiles along the radial coordinate (distance from the axis of rotation). The different colors represent the various measurement times.*

BO + 15 % 1-octanol

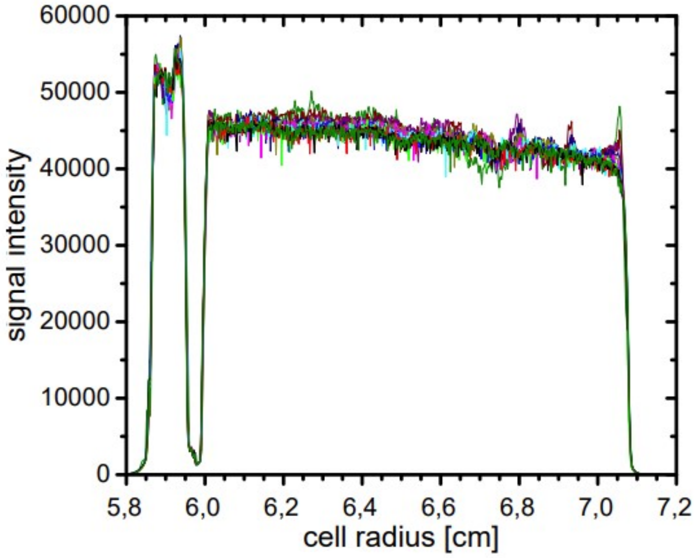

Figure 7: *SV-AUC measurement of the BD-BO system with 15 wt% 1-octanol using a constant rotor speed of 1000 rpm. It produces concentration profiles along the radial coordinate (distance from the axis of rotation). The different colors represent the various measurement times.*

BO + 20 % 1-octanol

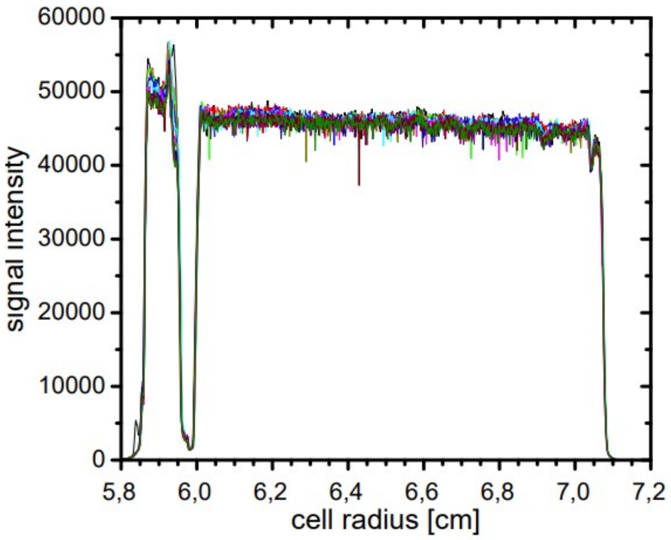

Figure 8: *SV-AUC measurement of the BD-BO system with 20 wt% 1-octanol using a constant rotor speed of 1000 rpm. It produces concentration profiles along the radial coordinate (distance from the axis of rotation). The different colors represent the various measurement times.*

Abbreviations

BHT	Butylhydroxytoluene
BO	Base oil
CFPP	Cold Filter Plugging Point
DIN	German Institute for Standardization
EN	European Standard
HFRR	High Frequency Reciprocating Rig
ISO	International Organization for Standardization
PUFA	Polyunsaturated Fatty Acids
SV-AUC	Sedimentation velocity-Analytical Ultracentrifugation
